# Transcriptome Profiling and Functional Validation of RING-Type E3 Ligases in Halophyte *Sesuvium verrucosum* under Salinity Stress

**DOI:** 10.3390/ijms23052821

**Published:** 2022-03-04

**Authors:** Fayas Thayale Purayil, Naganeeswaran Sudalaimuthuasari, Ling Li, Ruwan Aljneibi, Aysha Mohammed Khamis Al Shamsi, Nelson David, Martin Kottackal, Mariam AlZaabi, Jithin Balan, Shyam S. Kurup, Khaled Michel Hazzouri, Khaled M. A. Amiri

**Affiliations:** 1 Khalifa Center for Genetic Engineering and Biotechnology, United Arab Emirates University, Al-Ain P.O. Box 15551, United Arab Emirates; fayas.t@uaeu.ac.ae (F.T.P.); naganeeswaran@uaeu.ac.ae (N.S.); li.ling@uaeu.ac.ae (L.L.); raljenaibi@uaeu.ac.ae (R.A.); amshamsi@uaeu.ac.ae (A.M.K.A.S.); martin@uaeu.ac.ae (M.K.); zmaryam@uaeu.ac.ae (M.A.); jithinb@uaeu.ac.ae (J.B.); 2 Department of Integrative Agriculture, College of Food and Agriculture, United Arab Emirates University, Al-Ain P.O. Box 15551, United Arab Emirates; skurup@uaeu.ac.ae; 3 Center for Genomics and Systems Biology, New York University, Abu-Dhabi P.O. Box 129188, United Arab Emirates; drn2@nyu.edu; 4 Department of Biology, College of Science, United Arab Emirates University, Al-Ain P.O. Box 15551, United Arab Emirates

**Keywords:** *Sesuvium verrucosum*, salinity stress, E3 ligase, root transcriptome

## Abstract

Owing to their sessile nature, plants have developed a tapestry of molecular and physiological mechanisms to overcome diverse environmental challenges, including abiotic stresses. Adaptive radiation in certain lineages, such as Aizoaceae, enable their success in colonizing arid regions and is driven by evolutionary selection. *Sesuvium verrucosum* (commonly known as Western sea-purslane) is a highly salt-tolerant succulent halophyte belonging to the Aizoaceae family; thus, it provides us with the model-platform for studying plant adaptation to salt stress. Various transcriptional and translational mechanisms are employed by plants to cope with salt stress. One of the systems, namely, ubiquitin-mediated post-translational modification, plays a vital role in plant tolerance to abiotic stress and other biological process. E3 ligase plays a central role in target recognition and protein specificity in ubiquitin-mediated protein degradation. Here, we characterize E3 ligases in *Sesuvium verrucosum* from transcriptome analysis of roots in response to salinity stress. Our de novo transcriptome assembly results in 131,454 transcripts, and the completeness of transcriptome was confirmed by BUSCO analysis (99.3% of predicted plant-specific ortholog genes). Positive selection analysis shows 101 gene families under selection; these families are enriched for abiotic stress (e.g., osmotic and salt) responses and proteasomal ubiquitin-dependent protein catabolic processes. In total, 433 E3 ligase transcripts were identified in *S. verrucosum*; among these transcripts, single RING-type classes were more abundant compared to multi-subunit RING-type E3 ligases. Additionally, we compared the number of single RING-finger E3 ligases with ten different plant species, which confirmed the abundance of single RING-type E3 ligases in different plant species. In addition, differential expression analysis showed significant changes in 13 single RING-type E3 ligases (*p*-value < 0.05) under salinity stress. Furthermore, the functions of the selected E3 ligases genes (12 genes) were confirmed by yeast assay. Among them, nine genes conferred salt tolerance in transgenic yeast. This functional assay supports the possible involvement of these E3 ligase in salinity stress. Our results lay a foundation for translational research in glycophytes to develop stress tolerant crops.

## 1. Introduction

Plant growth and survival are influenced by numerous biotic and abiotic factors. Plants evolved complex and efficient molecular defense systems to cope with various environmental stresses that would otherwise hinder their growth and development [1]. Protein ubiquitination is one of the main post-translational regulation mechanisms in eukaryotes. It controls protein function through the addition of ubiquitin, a conserved 76 amino acid protein. In plants, ubiquitin-mediated protein degradation through the 26S proteasome system plays a major role in various plant developmental processes and responses to environmental cues [2,3]. In *Arabidopsis thaliana*, 6% of the protein-coding genes are reported to constitute the ubiquitin proteasome system (UPS), which underscores its importance in plants [4].

The process of ubiquitination is controlled by three enzyme groups: ubiquitin-activating (E1), ubiquitin-conjugating (E2), and ubiquitin-ligase (E3) enzymes. The first step in the ubiquitin-mediated proteasome system (UPS) is the E1 activation of ubiquitin through ATP-dependent thioester bond formation between a ubiquitin molecule and E1 cysteine residue(s). In the second stage, the ubiquitin is transferred to an E2 cysteine residue. Subsequently, the ubiquitin molecule is transferred to the target protein by an E3 ubiquitin ligase [5]. The target protein recognition and specificity are primarily controlled by E3 ligases. The polyubiquitin chain is assembled with ubiquitin lysine residues that direct the targeted protein to either the UPS or other endocytosis and autophagy programs [6,7,8].

E3 ubiquitin ligases are the most abundant ubiquitination enzymes in Eukaryotes. For instance, rice, maize, and *Arabidopsis* genomes each harbors over 1000 genes encoding E3 ligases, and their abundance is related to the target proteins specificity [9]. Based on conserved domains, function, and specificity, the E3 ligases are classified into three major groups: single and complex RING (Really Interesting New Gene)-type, Homology to E6-Associated Carboxyl-Terminus (HECT)-type, and U-box-type (U-box) [9]. In plants, the RING-type E3 ligase is the largest class of E3 ligases, and their abundance represent their diverse function in numerous biological processes [9].

Because ubiquitination plays a critical role in plant stress tolerance, many studies have focused on the E3 ligases, especially the RING-type E3 ligases because of their abundance. The RING-type E3 ligase contains a conserved 40–60 residues of cysteine-rich zinc-finger domain (RING-finger Domain) [10,11]. For instance, in *Arabidopsis*, overexpression of SDIR1 (Salt-and Drought-Induced RING Finger 1) results in salt and ABA hypersensitivity, enhanced stomatal closure, and drought tolerance [12,13]. Similar results were reported in transgenic rice by overexpressing rice SDIR1 [14]. However, E3 ligase regulation of abiotic stress responses and their target proteins remain poorly understood. Thus, it is imperative to characterize them in a systematic way.

*Sesuvium verrucosum*, a halophyte flowering plant of the Aizoaceae family, also known as Western sea-purslane, is a drought and salt-tolerant perennial found in the Arabian Peninsula as well as in the tropical and subtropical regions of the Americas. *S. verrucosum* can grow in saline and alkaline coastal and inland habitats [15]. *Sesuvium* is used as food, as well as fodder in South America and Southeast Asia. In addition, it has been widely used for environmental protection, such as sand dune fixation, desert greenification, and landscaping [16]. A recent study showed the benefit of using the salt-tolerant nature of *S. verrucosum* for rapid phytodesalination of saline soil to promote the growth of glycophyte *Zea mays* [17].

The molecular mechanisms underlying *S. verrucosum’s* drought and salt tolerance are poorly understood. The present study provides the first transcriptome analysis in *S. verrucosum* generated under salinity stress. We characterized the differential expression of genes under salinity stress with a major emphasis on E3 ligases. We used selection, orthology, and expression analysis, followed by functional validation of E3 ligases in transgenic yeast to provide valuable information relating to the salt tolerance mechanisms in *S. verrucosum*. We highlight several single RING-finger E3 ligase genes that will lay the foundation for the future development of stress-tolerant crops.

## 2. Results

### 2.1. RNA-Seq Quality Check, De Novo Assembly, and Annotation

We generated ~307 million 101 bp PE RNA-Seq reads with an Illumina NovaSeq 6000 from *Sesuvium verrucosum* root tissue that constitutes three control samples, three salt treatment early samples, and three salt treatment late samples (Table S1). More than 97% of the sequencing reads had a Phred score > Q20. After rRNA contamination removal, adapter trimming, and low-quality read trimming, we retained 233 million paired-end (PE) reads. De novo assembly of the trimmed reads yielded 301,627 transcripts (GC%: 43.1; N50: 2373) (Table S2). The assembly quality of the transcriptome was further confirmed using BUSCO (~99.5% of single copy plant-specific orthologous genes) and read alignment back to assembled transcripts (98%). Further, assembled transcripts were clustered and 195,255 transcripts were generated (Table S2). By similarity search (BLAST) of these clustered transcripts against the UniPrto-trEMBL protein database, the bacterial and fungal related transcripts were removed manually from the cluster. Blob plots were generated to highlight the contaminants found in the assembly (Figure S1). After removing the contamination, we retained 131,454 final transcripts (total size ~207 Mb, N50 = 2531, G + C% = 39.5, length range= 295–29,964 bp, average transcript length = 1579 bp). The BUSCO analysis predicted ~99.3% (single copy plant-specific orthologous genes) expressed genes from the final assembly. The read alignment of individual samples showed that 92% of reads aligned back to the final transcriptome (Table 1).

**Figure 1:**
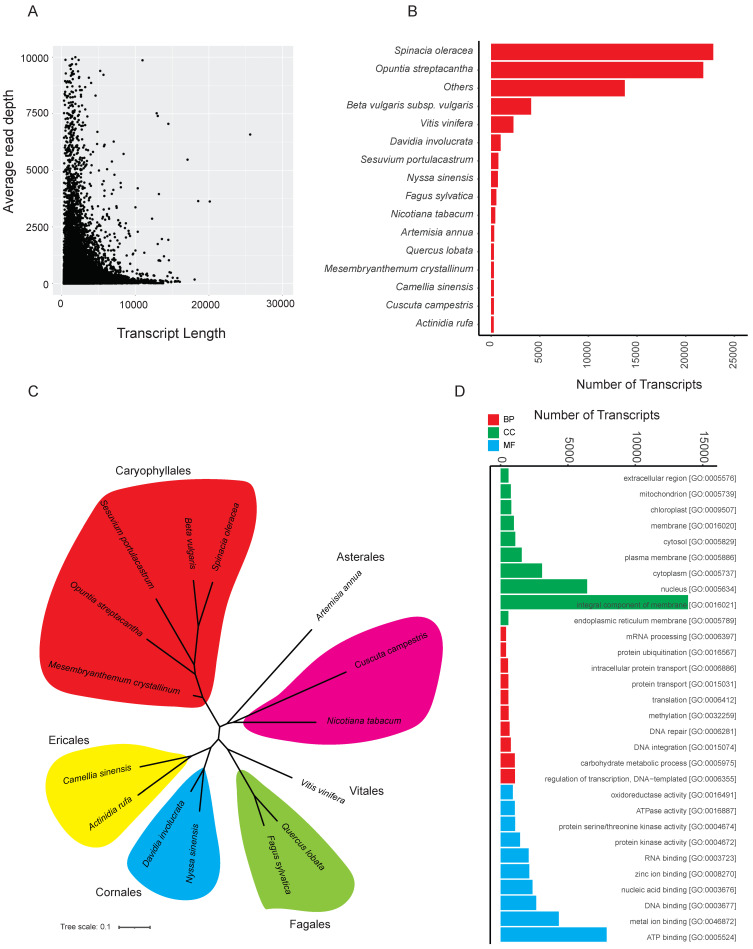
*Sesuvium verrucosum* de novo transcriptome assembly and quality check. (**A**) Distribution of average read depth relative to transcript length. (**B**) Bar graph showing similarity of *Sesuvium verrucosum* transcripts with plant (top 15 plants) database using Blast; “Others” represents remaining plant species. (**C**) Unrooted tree shows the phylogenetic relationship between the top 15 plants. (**D**) Ontology match of the *Sesuvium verrucosum* transcripts to GO term at biological (BP), molecular (MF), and cellular (CC) level.

A second quality check was performed by extracting the average read depth relative to transcript length (Figure 1A) to ensure that the majority of transcripts were represented. The transcripts were similarity searched against UniProt-trEMBL, UniProt-Plant, and *Arabidopsis thaliana* proteome databases; 69,826 (~53%), 69,816 (~53%), and 62,922 (~48%) transcripts were annotated in *S. verrucosum* root transcriptome from each of the respective databases (Table S2). *S. verrucosum* transcripts showed high similarity (as observed from top BLAST hits) with *Spinacia oleracea* (22,830), *Opuntia streptacantha* (21,804), *Beta vulgaris* subsp. *Vulgaris* (4109), and *Vitis vinifera* (2296) (Figure 1B). The relationships between the identified top hit plant species are shown in Figure 1C. We were able to identify Gene Ontology (GO) IDs in 53,575 (~41%) assembled transcripts. We found 42,432 Biological Process (BP) GO ID’s, 77,656 Molecular Function (MF) GO IDs, and 45,556 Cellular Compartment (CC) GO IDs from the transcriptome (Table S3). The most abundant GO IDs found in the transcriptome are shown in Figure 1D.

### 2.2. Positive Selection and Ortholog-Based Analysis

**Figure 2:**
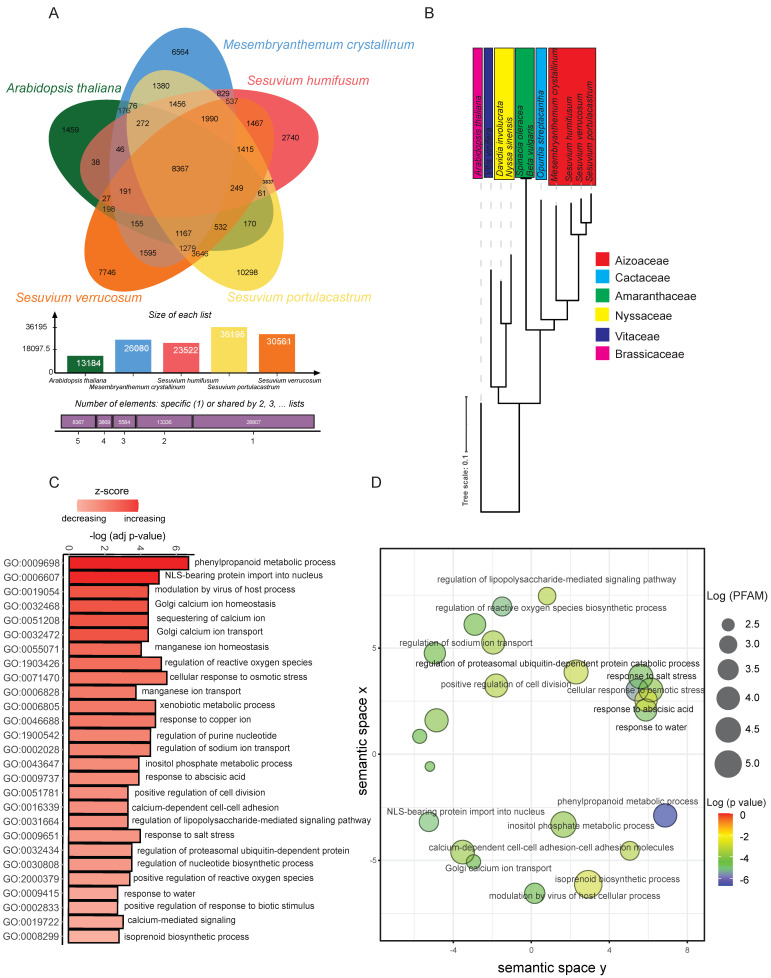
Positive selection and orthology analysis. (**A**) OrthoVenn2 analysis of Aizoaceae family: *Sesuvium verrucosum*, *Sesuvium portulacastrum*, *Sesuvium humifusum*, *Mesembryanthemum crystallinum*, and the outgroup *Arabidopsis thaliana*. (**B**) Single gene copy phylogenetic relationship of the Aizoaceae family with other related and distant ones using OrthoFinder. (**C**) PFAM enrichment of the positively selected gene families in Sesuvium identified using Fustr. Z-score reflects the strength of overlapped PFAMs and the *p*-value adjusted accounting for multiple hypotheses is for significance. (**D**) Revigo semantic analysis using the GO term of the positively selected genes. Log (PFAM size): logarithm of PFAM size and Log (*p* value): logarithm of *p* value significance.

We performed an orthology analysis of the *S. verrucosum* transcriptome with other species (*S. portulacastrum*, *S. humifusum*, and *M. crystallinum*) of Aizoaceae and *Arabidopsis* as an outgroup (Figure 2A). This analysis revealed 59,963 clusters, 59,543 ortholog clusters containing at least two species, and 420 single-copy gene clusters (Figure S2). The five species shared 8367 orthogroups (Figures S2 and S3).

A phylogenetic analysis was performed with OrthoFinder [18], and the results confirmed the relationship of the Aizoaceae species with its relative species of the family Cactaceae and Amaranthaceae (Figure 1B and Figure 2B). The FUSTr positive selection analysis identified 101 gene families under positive selection (Table S4). We examined the protein family (PFAM) enrichment of these genes using dcGO [19] and found that GO terms related to abiotic osmotic and salt stress response, such as response to abscisic acid, response to salt stress, and response to water, were uniquely enriched in the salt treatment groups (Figure 2C). Interestingly, the GO term [32434] ‘regulation of proteasomal ubiquitin-dependent protein catabolic process’ was also enriched. The single-RING E3 ligase (SIAH1) is a representative gene linked to this GO term. We used the GO terms in Revigo analysis [20] to draw a closer relationship using hygrometric semantic plots of the different GO terms (Figure 2D). Abiotic stress terms clustered closer to each other than other GO terms, including the regulation of proteasomal ubiquitin-dependent protein catabolic process.

### 2.3. Classification and Comparison of E3 Ligase Abundance

**Figure 3:**
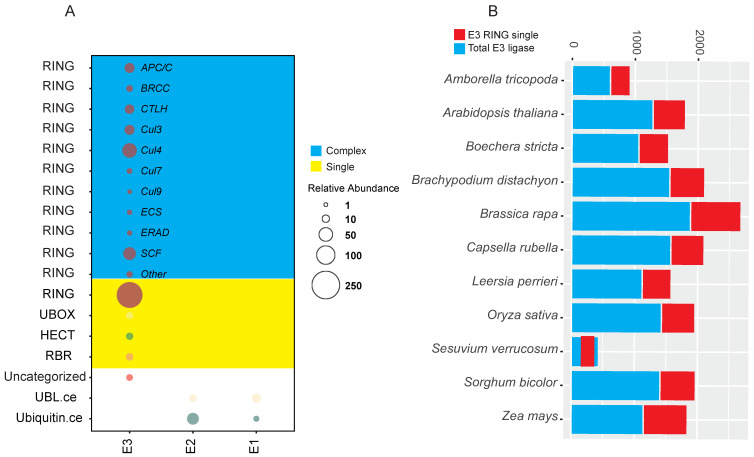
Classification and comparison of E3 ligase. (**A**) Abundance of E3, E2, E1 ligase in complex and single form. (**B**) Comparison of E3 single-RING ligase count relative to the total number of transcripts in different species (*Amborella tricopoda*, *Arabidopsis thaliana*, *Boechera stricta*, *Brachypodium distachyon*, *Brassica rapa*, *Capsella rubella*, *Leersia perrieri*, *Oryza sativa*, *Sesuvium verrucosum*, *Sorghum bicolor*, *Zea mays*).

Expressed metabolic pathway enzymes were identified using KEGG-KAAS. A total of 11,484 (~9%) transcripts associated with various metabolic pathways were identified (Table S5) from the root transcriptome of *S. verrucosum*. From the KEGG annotation, 632 transcripts that were involved in Ubiquitin system (ubiquitin-activating enzyme (E1), ubiquitin-conjugating enzyme (E2), E3 ubiquitin-ligases, deubiquitinating enzymes, and Ubiquitins and ubiquitin-like proteins) were identified (Table S6). Components of the ubiquitin system described herein are based on the KEGG-KO IDs retrieved in the KEGG-KAAS analysis. These results showed that E3 ligase enzymes (433 transcripts) are predominant in the *S. verrucosum* root transcriptome compared to other classes of ubiquitin enzymes. We further classified E3 ligase enzymes into different sub-categories and found that single RING-finger E3 ligase enzymes (212 transcripts) and multi-subunit RING-finger E3 ligase enzymes (186 transcripts) were predominantly expressed. Other types of E3 ligases were also found, including atypical E3 ligases, HECT-type E3 ligases, U-box type E3 ligases, and UBL E3 ligases (Figure 3A). Furthermore, we compared the number of single RING-finger E3 ligases with ten different plant species (Figure 2B). The E3 ligase-based phylogenetic relationship between the ten selected plant species is shown in Figure S4. Overall, the single RING-type E3 ligase showed ~37–50% abundance compared to other E3 ligase classes in all the species compared (Figure 3B).

### 2.4. Differential Gene Expression Analysis

**Figure 4:**
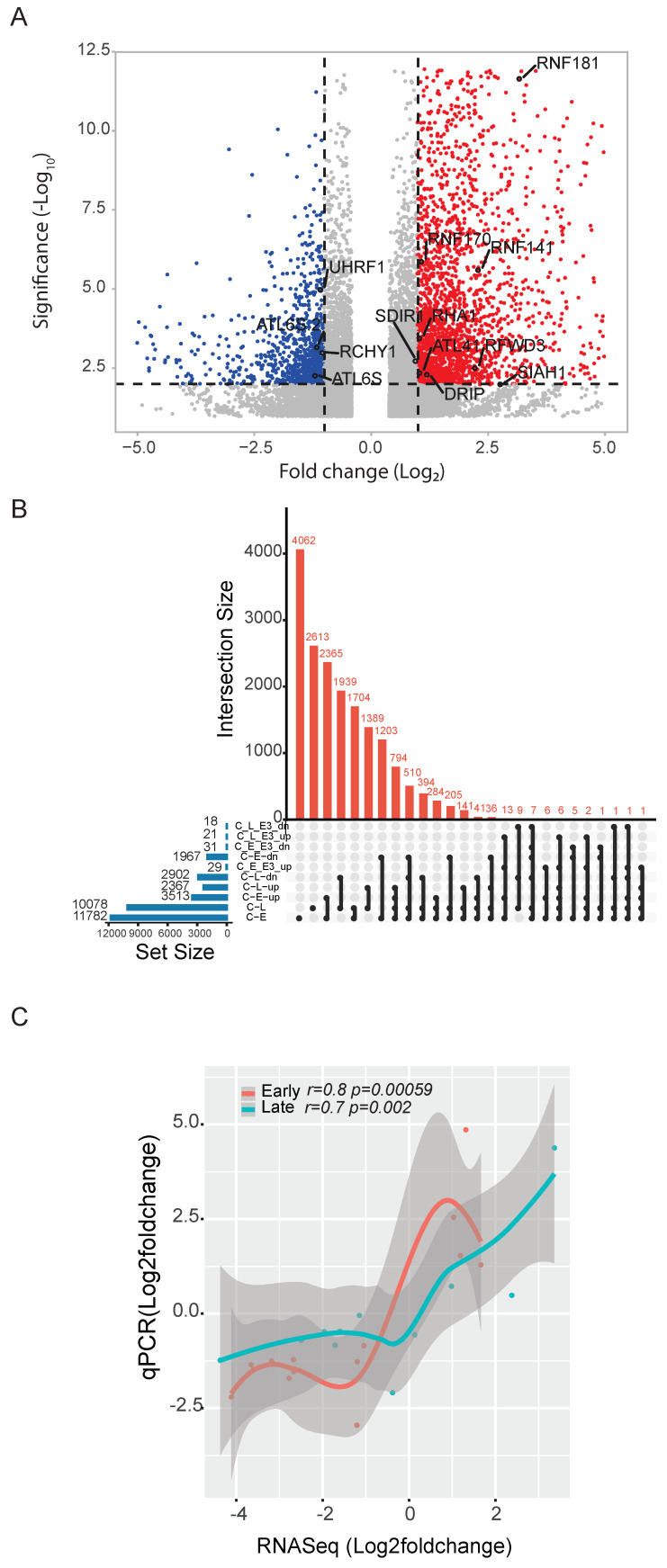
Differential expression of genes and E3 ligase at early and late stages of stress. (**A**) Volcano plot depicting log2 fold change of upregulated (red) and downregulated (blue) genes. E3 single-RING ligase upregulated and downregulated are highlighted in black. (**B**) UpsetR plot showing the intersection of differentially expressed genes with each other as well as E3 ligase in early and late salt stress (C: Control, E: Early, L: Late, E3: E3 ligase, up: Upregulated and dn: Downregulated). (**C**) Correlation of early and late RNA-Seq to qRT-PCR of 14 randomly selected up- and downregulated genes.

Differential gene expression analysis showed 3513 significantly upregulated genes (log2 fold change ≥ 1 and *p* < 0.05) and 1967 downregulated genes (log2 fold change ≤ −1 and *p* < 0.05) in samples from the ‘early’ salt treatment group. Comparison of the control with the ‘late’ salt treatment group showed 2367 upregulated genes and 2902 downregulated genes. The E3 ligase differential expression was limited to 13 genes at that magnitude change (Figure 4A). This is highlighted in the UpsetR intersection plot of different transcript expressions at early and late stages, including E3 ligases, which is represented at the tail of the distribution (Figure 4B). The majority are significantly (*p*-value < 0.05) represented by the single RING-type E3 ligase (Figure 4A,B, Table S6).

To confirm the RNA-Seq-based differential gene expression results, we carried out qPCR experiments for 14 genes. We selected seven single-RING E3 ligase genes and seven highly/moderately expressed other genes for the RT-qPCR analysis (Table S7). The correlation analysis between the RNA-Seq results and qPCR results showed a good correlation (*p*-value < 0.001; r = 0.8 (Early) and r = 0.7 (Late)) between the studies (Figure 4C). Some of the E3 ligase transcripts were upregulated in the early stage of salinity stress, which decreased significantly under prolonged salinity stress. Oxidative stress-responsive enzymes such as 2-alkenal reductase and peroxidase 1 were also upregulated (Table S7).

### 2.5. Yeast Functional Analysis of E3 Single-RING E3 Ligases

**Figure 5:**
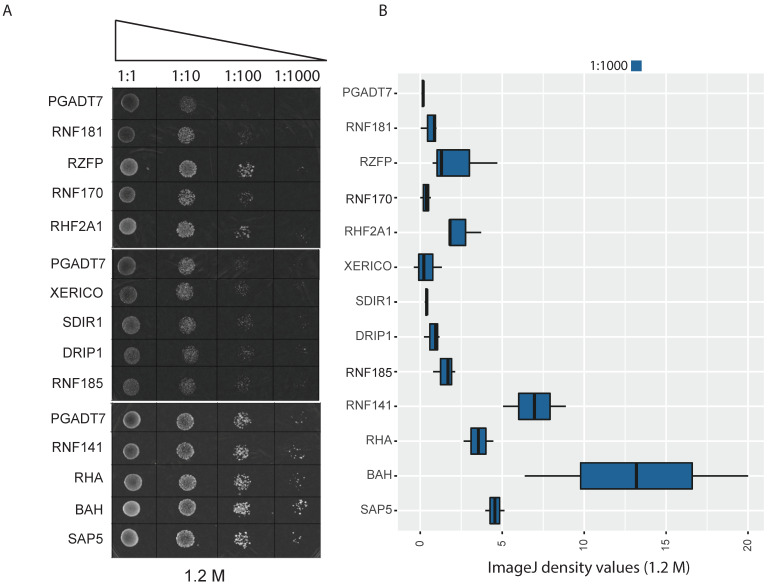
Yeast functional drop test assay. (**A**) Three plates representing the yeast-based functional validation of 12 selected E3 ligase genes and empty vector control (pGADT7) under 1.2 M salt concentration. The dilution factors used in this study is given on the top of the panel (column wise). (**B**) Image J image quantification of the drop density as a proxy for growth in replicates of the 1.2 M compared to the control.

To determine if the differentially expressed single RING E3 ligase genes affect salinity tolerance, we selected 12 genes whose transcripts were upregulated upon salinity stress for a transgenic yeast system to explore their functionality. The yeast functional analysis was performed using a spot assay on medium containing 1.2 M NaCl. Yeast transformed with orthologs of BAH, SAP5, RHA, RNF141, RNF185, DRIP1, RHF2A1, RZFP, and SDIR1 show higher growth under salinity stress as compared to an empty vector control (Figure 5A). This result is consistent with the image analysis of colony growth density (Figure 5B). While many of the selected E3 ligases have not previously been linked to abiotic stress tolerance, several studies have shown that SAP5, SDIR, and DRIP play important roles in regulating the abiotic stress response. The yeast growth assay supports the potential involvement of these E3 ligases in mediating salinity stress response. Further study is needed to elucidate their function in plant stress response.

## 3. Discussion

Soil salinity is considered a major threat to plant growth and productivity. To overcome the inhibitory effects of salt, plants have evolved complex physiological and biochemical adaptations. Lately, Halophytes have gained much attention due to their remarkable potential to survive under salinity stress. Their ability not only to survive but also to complete their life cycle make them ideal models to unravel the molecular mechanisms of salinity stress adaptation. In this study, we generated the first high-depth RNA-Seq data for the halophyte *S. verrucosum* under salinity stress. The de novo assembly generated approximately 131,454 transcripts, which represent ~99.3% single copy plant orthologous genes, indicating a high-quality transcriptome assembly compared to other halophytes already reported [21,22]. Approximately 53% of the genes were annotated using the UniProt-trEMBL database.

Based on the evidence of large-scale adaptive radiation in the Aizoaceae family, we investigated the rapidly evolving gene families in *S. verrucosum.* Our positive selection results identify gene families implicated in abiotic stress tolerance. These families include genes coding for E3 ligase. Our characterization of *S. verrusosum* E3 ligases revealed RING, UBOX, HECT, and RBR classes. Among the different classes of E3 ligases, the RING-type, especially the single RING-type E3 ligases, are reported to play an important role in tolerance to abiotic stresses, including salinity and drought [23]. The RING-type E3 ligases are the most abundant in *S. verrucosum*, and they are comparable to the number of RING-type reported in peach, grape, soybean, poplar, and maize [24]. Approximately 48% of the identified E3 ligases transcripts in *S. verrucosum* are the RING-type. In the *Arabidopsis* genome, nearly 36% of the E3 ligases are RING-type, which accounts for almost 2% of the predicted proteins [4,25]. Their abundance suggests important and diverse roles of the RING-type E3 ligases in plant growth and development.

In the present study, yeast-based functional characterization of differentially expressed E3 ligases provided evidence of single RING-type E3 ligases for salinity tolerance enhancement in *S. verrucosum*. Together with orthologs of known E3 ligases such as XERICO and SDIR, we tested several E3 ligases in our transgenic yeast growth assays to elucidate their role in salinity tolerance. Interestingly, yeast colonies transformed with novel BAH-type, RNF 141-like, and RHA-type E3 ligases showed an increased tolerance to salinity stress, indicating their possible role in stress tolerance (Figure 5A,B).

Stress response mechanisms in plants are classified into two major groups: abscisic acid (ABA)-dependent and ABA-independent mechanisms. In ABA-dependent stress responses, the expression of stress-responsive genes is regulated by cis-acting ABA-responsive elements (ABRE) and multiple transcription factors [26]. Whereas in the ABA-independent mechanism, dehydration responsive element (DRE) and DREB2A transcription factors are deployed for stress tolerance. The expression level of DREB2A is regulated by two RING-type E3 ligases named DRIP1 and DRIP2 [27]. Studies suggest that DRIP E3 ligases act as negative regulators of DREB2A-mediated drought stress responses in *Arabidopsis*. Overexpression of DRIP1 resulted in significant delays in the expression of DREB2A-dependent drought-responsive genes. On the other hand, Stress-Associated Protein 5 (SAP5), a A20/AN1-type E3 ligase, functions as a positive regulator of stress tolerance by promoting the degradation of DRIP proteins [28,29]. Our functional analysis showed both *S. verrucosum* DRIP and SAP5 orthologs positively regulated salt tolerance in yeast (Figure 5); whereas expression data showed the upregulation of a DRIP ortholog in the early stage of the salinity stress, but this expression declined after prolonged exposure to salt stress. Furthermore, the expression of a SAP5 ortholog (TRINITY_DN3310_c0_g1_i1) is significantly (*p*-value: 0.0060) induced throughout salinity stress in *S. verrucosum*, suggesting a possible interaction between DRIP and SAP5 orthologs in *S. verrucosum.*

XERICO, a RING-type E3 ligase, is reported to be involved in stress tolerance through an ABA-dependent pathway [30]. In *Arabidopsis*, overexpression of XERICO triggered the upregulation of AtNCED3, a key enzyme in ABA synthesis, eventually leading to elevated endogenous ABA levels and promoting increased tolerance to drought stress [30]. Recent studies have also shown the overexpression of AtXERICO in rice as well as the overexpression of ZmXERICO1 in maize conferred improved tolerance to drought and salinity stress [31,32]. The expression of a XERICO ortholog in *S. verrucosum* was highly induced in early stages of salinity stress, which implies its role as a ‘first-responder’ in *S. verrucosum* to salinity stress. Interestingly, the transcript levels of several genes in the ABA synthesis pathway, including genes encoding for NCED enzymes, were upregulated in our data. These findings suggest functional conservation of XERICO genes in monocots and dicots.

Another RING-type E3 ligase, SALT-AND DROUGHT-INDUCED RING FINGER 1 (SDIR1), was reported to play a key role in the ABA-mediated stress signaling pathway by controlling transcriptional regulation of ABI3 and ABI5 TFs [12]. Overexpression of the SDIR1 gene in *Arabidopsis* yielded increased tolerance to drought stress [12]. The SDIR1 gene also regulates the ABA-depended salt stress responses in *Arabidopsis* [13]. Similar findings were reported in rice by the overexpression of OsSDIR1 [14]. Recently, SDIR1-INTERACTING PROTEIN1 (SDIRIP1), a negative regulator of ABA signaling, was identified as the substrate for SDIR1 gene. We observed a significant upregulation of SDIR1 transcript (TRINITY_DN6910_c0_g1_i54, *p*-value: 0.0018) throughout the early and late phases of salinity stress (Figure 4A, Table S8). The yeast spot assay test also confirms the possible role of the SDIR1 gene in the salinity tolerance of *S. verrucosum* (Figure 5).

In plants, Seven In Absentia (SINA) is a group of E3 ligases involved in many biological processes, including autophagy and drought responses. In *Arabidopsis*, five SINA members were reported, SINAT1-5 (SINA of *Arabidopsis* thaliana 1–5). SINAT5 is the first of the SINA family to be characterized, and it is shown to negatively regulate auxin dependent lateral root development in *Arabidopsis* [33]. Whereas in rice, a SINAT5 ortholog, *Oryza sativa* Drought-induced SINA protein 1(OsDIS1) acts as a negative regulator of drought stress by altering transcriptional regulation of stress responsive genes [34]. Overexpression of the AtSINAT2 gene led to drought tolerance by increasing stomatal closure, resulting in water loss mitigation in *Arabidopsis* [35]. In *S. verrucosum*, a SINA E3 ligase transcript closely related to *Arabidopsis* SINAT2 was significantly upregulated under salinity stress (TRINITY_DN1735_c0_g1_i19) (*p*-value: 0.0098) (Figure 4A). The expression level was elevated throughout the salinity stress experiment (Table S7). This indicates that SINA-type ligases may also regulate salinity tolerance of *S. verrucosum*.

In conclusion, this study provides valuable insight into the molecular programming of E3 ligases in halophytes. In addition to the above-mentioned known E3 ligase orthologs, we have also identified the differential expression of several E3 ligases previously unknown to respond to abiotic stress in our salt treatments. Many of these new salt-responsive E3 ligases increased salinity tolerance in our transgenic yeast growth assay and provide the basis for further study of salinity tolerance and translational research for crop improvement.

## 4. Materials and Methods

### 4.1. Sample Preparation, RNA Isolation, and Sequencing

Salinity stress experiments of *Sesuvium verrucosum* were performed in a hydroponic system. *S. verrucosum* cuttings containing three nodes from the apical shoots were grown in 1/10 strength Murashige and Skoog solution for five weeks for rooting. The rooted plants were treated with 300 mM NaCl dissolved in 1/10 strength Murashige and Skoog liquid medium. The root samples were collected after the stress treatment at different time points and were labeled as ‘Early’ and ‘Late’ samples. The ‘Early’ samples include the pooled samples collected at 4 h and 24 h of salinity treatment, whereas the ‘Late’ samples represent pooled samples collected on the 3rd and 7th day of stress. The control root samples were collected from cuttings grown in 1/10 strength Murashige and Skoog liquid medium without salt. Three biological replicates were included for each sample set. The collected samples were flash-frozen immediately in liquid nitrogen and stored at −80 °C.

Total RNA was extracted from the collected root samples using a modified CTAB method [36]. The samples were homogenized in liquid nitrogen using a mortar and pestle. The homogenized root samples (100 mg) were added to 5 mL of CTAB (Cetyl trimethylammonium bromide) buffer (2% *w*/*v*), 2% (*w*/*v*) PVP (Polyvinylpyrrolidone K30), 100 mM Tris-HCl, pH 8.0, 25 mM EDTA (Ethylenediaminetetraacetic acid), 2.0 M NaCl, 500 mg/L Spermidine (Sigma), and 2% (*v*/*v*) β-Mercaptoethanol (added just before extraction). Samples were incubated at 65 °C for 15 min with occasional mixing. After centrifugation at 12,000 rpm, an equal volume of *Chloroform: Isoamyl* alcohol (24:1) was added to the supernatant and mixed. The RNA was precipitated using 10 M LiCl, washed two times with 70% ethanol, air-dried, and dissolved in nuclease-free water. The quantity of the RNA was determined by Nanodrop 2000 (Thermo Scientific, Waltham, MA, USA), and the quality was checked using agarose (1% *w*/*v*) gel electrophoresis. Illumina compatible RNA-Seq libraries were prepared from the RNA using TruSeq Stranded Total RNA LT Sample Prep Kit and sequenced in an Illumina NovoSeq 6000 (101 base pair (bp) paired-end (PE) reads).

### 4.2. Transcriptome Analysis

The sequencing reads were quality checked with FastQC [37]. Transcriptome reads were then corrected using a k-mer-based approach with Rcorrector [38]; uncorrectable (k-mer error) reads were removed from the transcriptome using TranscriptomeAssemblyTools (https://github.com/harvardinformatics/TranscriptomeAssemblyTools, accessed on 29 April 2021). The adapter and low-quality regions found in the reads were removed using TrimGalore v.0.5.0 (https://www.bioinformatics.babraham.ac.uk/projects/trim_galore/, accessed on 29 April 2021). The trimmed reads were again aligned against the SILVA v.138 rRNA database [39] using Bowtie2 v. 2.3.5.1 [40], and the possible rRNA contaminations were removed. Transcriptome read normalization and de novo transcriptome assembly were carried out using the Trinity v2.12.0 (--seqType fq --max_memory 900 G --samples_file sample.txt --min_contig_length 300 --SS_lib_type RF --CPU 60) program [41]. The transcriptome assembly quality was initially confirmed by the transcriptome read alignment back to the assembled transcripts using Bowtie2. Furthermore, assembled transcripts were clustered using CD-HIT (>85% similarity) [42]. The quality of the final transcriptome assembly was again confirmed with BUSCO (db: viridiplantae_odb10) [43].

### 4.3. Differential Expression, Functional Annotation, and Characterization of E3 Ligases

Differential gene expression (DGE) in control and salt treatments was analyzed with DeSeq2 [44]. Differences in expressed genes were denoted as significant at *p* < 0.05 and log2 fold change ≥ 1 or log2 fold change ≤ −1. Transcript sequences were searched against the UniProt bacteria database using the Basic Local Alignment Search Tool (BLAST) [45], and the identified bacterial transcript contaminations were removed from the root transcriptome. Transcripts were then aligned against the entire UniProt protein, UniProt-Plant, and *Arabidopsis thaliana* proteome databases using BLAST (e-values: 10^−5^). The GO terms associated with the transcripts were mined from the UniProt database based on the BLAST similarity search. Assembled transcripts were searched against the KEGG pathway database [46] using the KAAS tool [47] and possible metabolic pathway genes were identified. Based on the KAAS results, different types of ubiquitination enzymes (E1, E2, and E3 ligase) were identified, and the identified enzymes were manually curated for the characterization of different classes of E3 ligases.

### 4.4. Positive Selection and Ortholog-Based Analysis

We generated ortholog cluster relationships using OrthoVenn2 [48] as well as OrthoFinder [18] to build a phylogenetic relationship among close and distant families. Protein-coding genes under positive selection were detected with FUSTr [49], a pipeline that performs branch-site tests on ortholog genes. *Sesuvium portulacastrum*, *Sesuvium humifusum*, and *Mesembryanthemum crystallinum* RNA-Seq data were downloaded from NCBI-SRA (SRX3423937, ERR2040193, SRX9647213), and de novo assembly was performed in Trinity [41] in a similar way as *Sesuvium verrucosum*. For FUSTr, the entire transcriptome of *S. verrucosum* and *S. portulacastrum* were used as input, and downstream pipeline was used for orthology and sequence alignment. Highly diverged or incomplete orthologs were removed from the analysis. FUSTr tests were carried out for positive selection in gene families irrespective of their taxonomic representation among other taxa. Isoforms were identified in the input sequences, and TransDecoder (https://github.com/TransDecoder/TransDecoder, accessed on 15 May 2021) was used to extract the best open reading frame for each transcript. Homology analysis was performed with Diamond [50] using peptide sequences, followed by grouping gene families using SiLiX [51]. Finally, alignments were carried out using MAFFT [52]. At least 15 amino acid sequences representing a gene family were tested at a site-specific level using the branch-site model implemented in FUBAR [53]. Genes under positive selection were compared to the DEG list to discern whether the positive selection and regulatory evolution are acting on the same genes.

### 4.5. RT-qPCR Validation

Real-time quantitative PCR analysis was performed to validate RNA-Seq expression patterns. The extracted RNA samples were converted to cDNA using a QuantiTect Reverse Transcription kit (Qiagen, Hilden, German) following the manufacturer’s instructions. Fourteen differentially expressed genes (DEGs) were selected for qPCR detection, and the PP2A gene was selected as an internal reference for the analysis. The qPCR analysis was performed using the SYBR Green PCR master mix (Applied Biosystems, Waltham, MA, USA). Three technical replicates were analyzed for each biological replicate. The relative expression pattern was calculated using the 2^−∆∆Ct^ method [54].

### 4.6. Functional Analysis in Yeast

Twelve differentially expressed E3 ligase genes were selected for functional characterization in yeast. The selected genes were cloned into the pGADT7 vector and transformed in *Saccharomyces cerevisiae* (strain Y187) using the Yeastmaker^TM^ Yeast Transformation System 2 (Takara Bio Inc., Kusatsu, Japan) and grown at 30 °C for three days. The presence of specific genes in transformed yeast colonies were confirmed using colony PCR. Spot assays of *S. cerevisiae* harboring the genes were performed on synthetic minimal media without Leucine (SD-Leu) plates as control, and SD-Leu containing 1.2 M NaCl as test. Transformed yeast cultures were grown in liquid SD-Leu until the culture reached an OD_600_ of 1, and then 10-fold serial dilutions up to 10^−4^ were spotted on each media [55]. Image analyses were performed according to Petropavlovskiy et al. [56]. The colony growth density of triplicates was compared to controls using the software ImageJ2 [57].

## Figures and Tables

**Table 1:** Summary statistics of *Sesuvium verrucosum* de novo transcriptome assembly.

	Primary Assembly	After Clustering	* Final Transcriptome
Total transcripts	301,627	195,255	131,454
Total bases	424,665,332	258,421,237	207,568,729
Read alignment %	98	95	92
BUSCO validation	~99.5% (96% complete)	~99.5% (96% complete)	~99.3% (95.8% complete)

* After removal of bacterial and fungal transcripts.

## Data Availability

The root transcriptome data generated during this study was deposited in NCBI-SRA database under the accession number: PRJNA762598.

## Supplementary Materials

The following supporting information can be downloaded at: https://www.mdpi.com/article/10.3390/ijms23052821/s1.
