# Supplementary material for: Transcriptome Profiling and Functional Validation of RING-Type E3 Ligases in Halophyte Sesuvium verrucosum under Salinity Stress
Source: Int J Mol Sci. 2022 Mar 4;23(5):2821. doi: 10.3390/ijms23052821 (PMC8911510; doi:10.3390/ijms23052821)
Supplement: Supplementary file 1 [file ijms-23-02821-s001.zip › Sup_Fig_captions.pdf]

Figure S1. Blob plot in roots of *Sesuvium verrucosum* showing in different colors the bacterial and fungal contamination, and how much they span in comparison to the actual Sesuvium genome using coverage and GC proportion.

Figure S2. OrthoVenn3 orthogroup clusters, showing in color what's shared and what's unique among the five different species in the study.

Figure S3. Heatmap of the number of shared orthogroup among the five different species in the study.

Figure S4. The phylogenetic relationship between *Sesuvium verrucosum* selected ten different plant species.
