# Supplementary material for: Transcriptome Profiling and Functional Validation of RING-Type E3 Ligases in Halophyte Sesuvium verrucosum under Salinity Stress
Source: Int J Mol Sci. 2022 Mar 4;23(5):2821. doi: 10.3390/ijms23052821 (PMC8911510; doi:10.3390/ijms23052821)
Supplement: Supplementary file 1 [file ijms-23-02821-s001.zip › Table_S2.pdf]

Supplementary Table 2. Transcriptome assembly statistics

	Primary assembly	After clustering	*Final transcriptome
Total transcripts	301627	195255	131454
Total bases	424665332	258421237	207568729
A+T percentage	56.80587367	56.51196616	60.2730491
G+C percentage	43.19412633	43.48803384	39.7269509
N %	0	0	0
Min sequence length	283	289	295
Max sequence length	29964	29964	29964
Average sequence length	1407.92	1323.51	1579.02
Median sequence length	817	756	1020
N50 length	2373	2221	2531
L50 number	53813	34519	25682
Sequence length 201 - 500	91999	63002	31003
Sequence length 501 - 1000	78751	53041	33895
Sequence length 1001 - 3000	94799	58668	47672
Sequence length 3001 - 5000	26284	15133	13986
Sequence length 5001 - 7000	6864	3838	3523
Sequence length 7001 – 10000	2379	1288	1159
Sequence length 10001 - .1MB	551	285	216
Read alignment %	98	95	92
BUSCO validation	~99.5% (96% complete)	~99.5% (96% complete)	~99.3% (95.8% complete)
Uniprot Trembl annotation			69826 (~53%)
Uniprot plant annotation			69816 (~53%)
KEGG annotation			11484 (~9%)
Arabidopsis thaliana annotation			62922 (~48%)
Interpro			52945 (~40%)
GO			53575 (~41%)

*- After contamination removal
