# Supplementary figures and images for: Transcriptome Profiling and Functional Validation of RING-Type E3 Ligases in Halophyte Sesuvium verrucosum under Salinity Stress
Source: Int J Mol Sci. 2022 Mar 4;23(5):2821. doi: 10.3390/ijms23052821 (PMC8911510; doi:10.3390/ijms23052821)

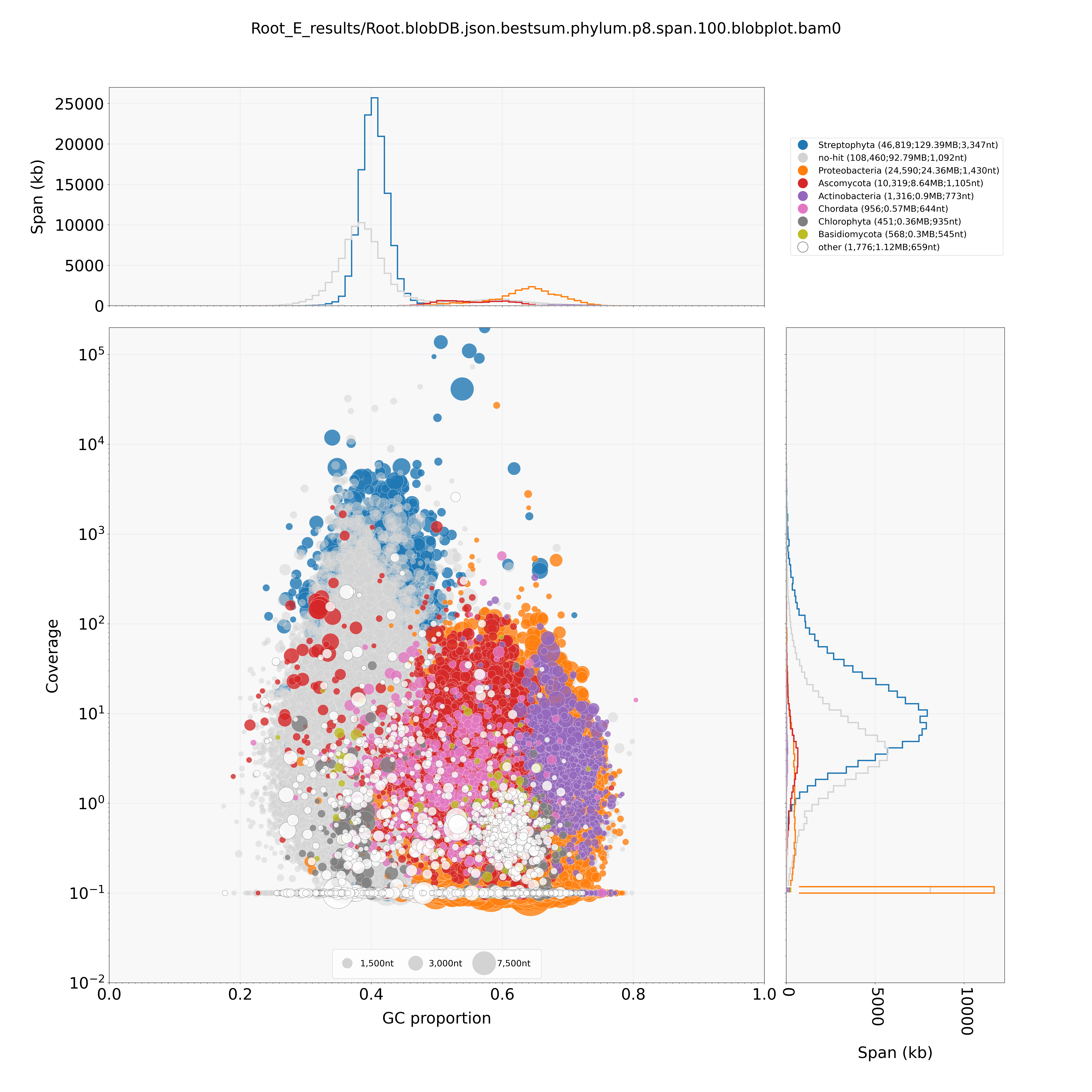

Supplement: Supplementary file 1 [file ijms-23-02821-s001.zip › Figure_S1.png]

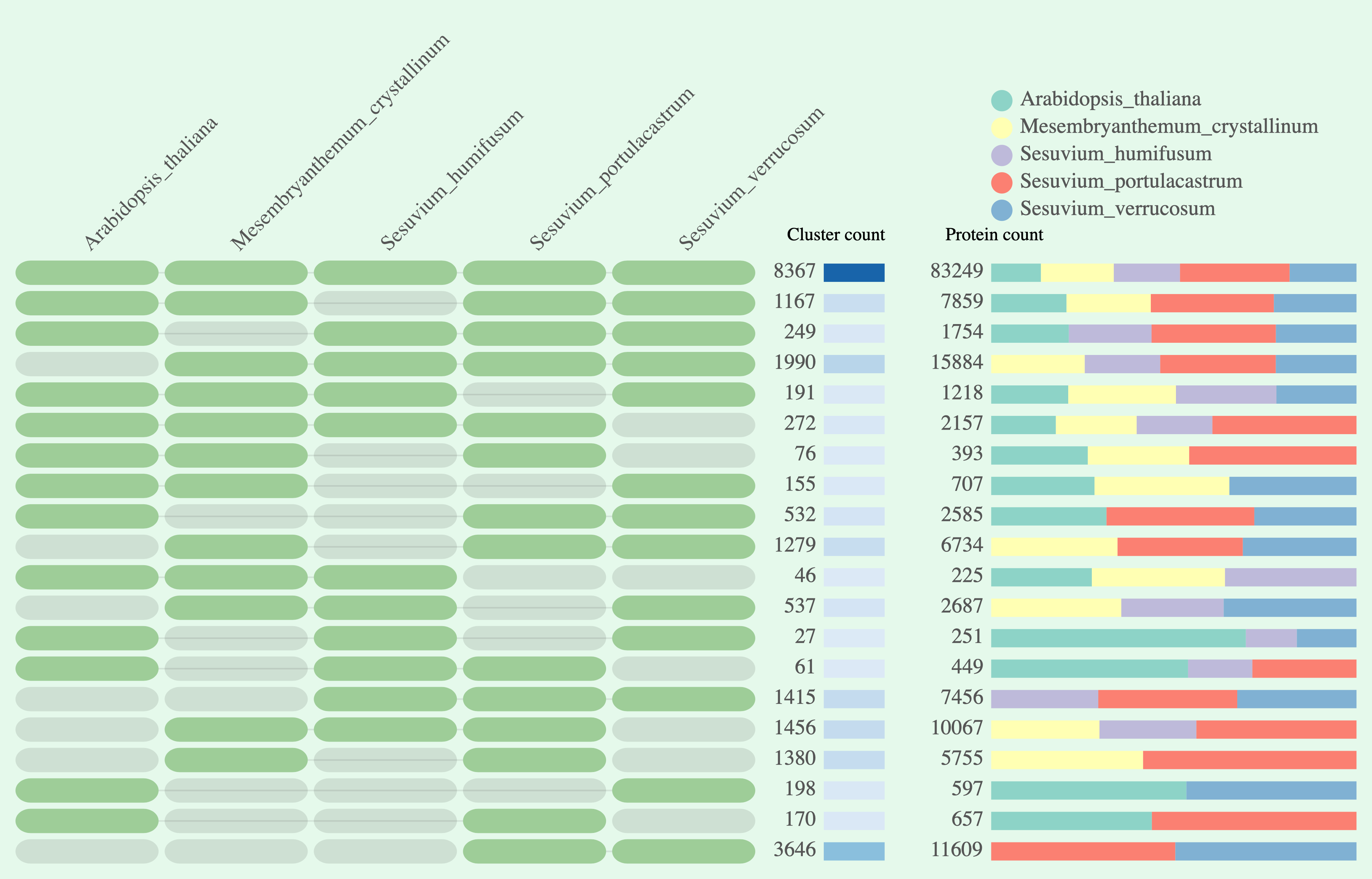

Supplement: Supplementary file 1 [file ijms-23-02821-s001.zip › Figure_S2.png]

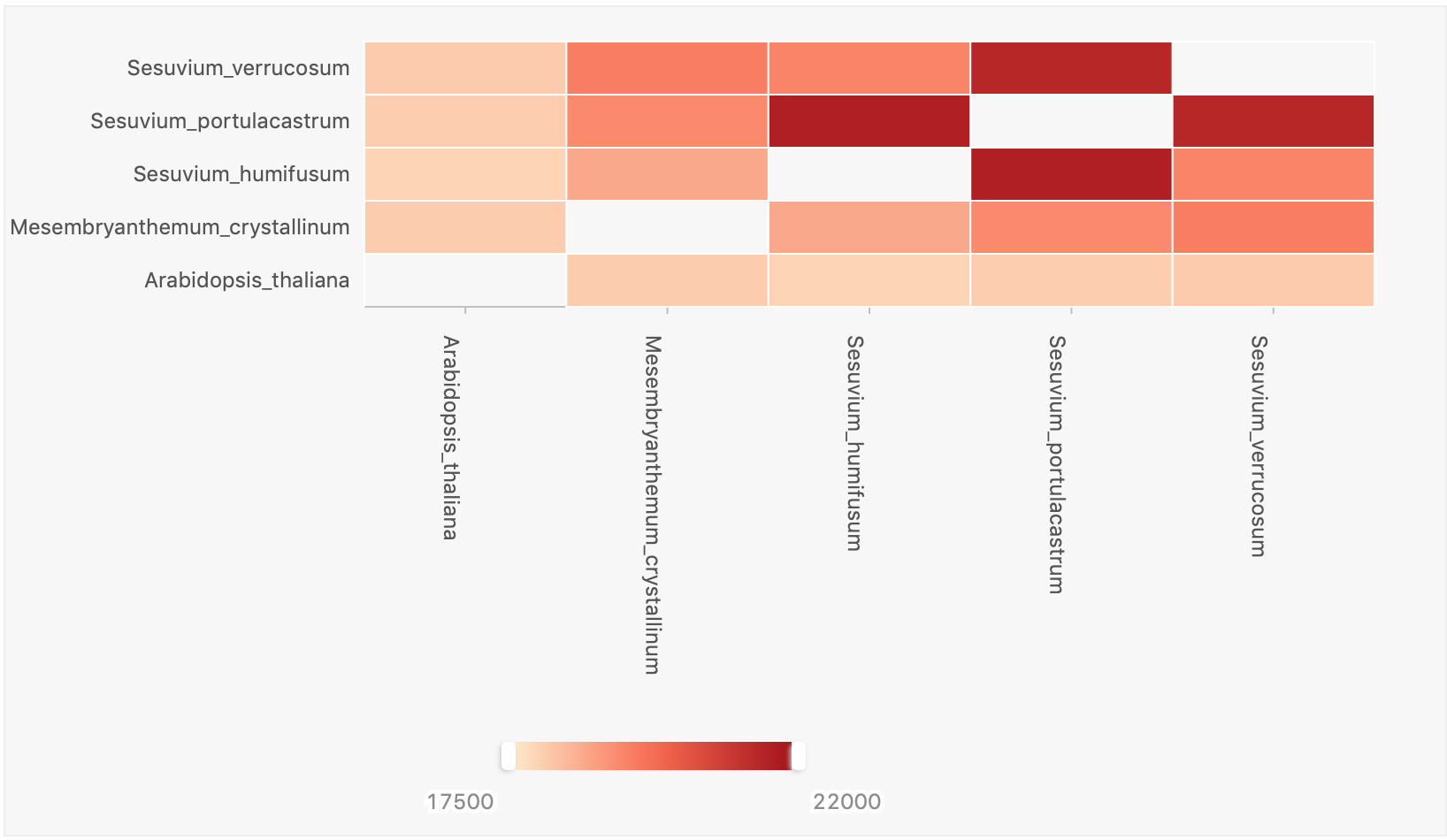

Supplement: Supplementary file 1 [file ijms-23-02821-s001.zip › Figure_S3.png]

Tree scale: 0.1

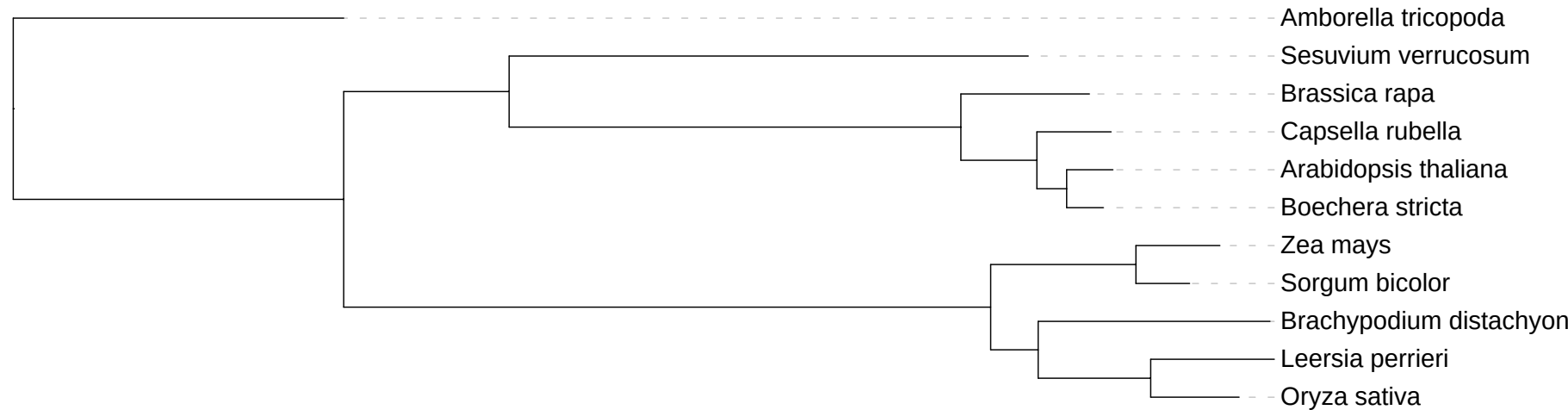

Supplement: Supplementary file 1 [file ijms-23-02821-s001.zip › Figure_S4.pdf]
